# Efficacy of a moxidectin/imidacloprid spot-on formulation (Advocate^®^) for the treatment of *Troglostrongylus brevior* in naturally infected cats in a field study in Greece

**DOI:** 10.1186/s13071-019-3760-9

**Published:** 2019-11-04

**Authors:** Anastasia Diakou, Simone Morelli, Dimitris Dimzas, Angela Di Cesare, Gioia Capelli, Chiara Parrinello, Matthias Pollmeier, Roland Schaper, Donato Traversa

**Affiliations:** 1 Laboratory of Parasitology and Parasitic Diseases, School of Veterinary Medicine, Faculty of Health Sciences, Aristotle University of Thessaloniki, 54124 Thessaloniki, Greece; 2 Faculty of Veterinary Medicine, University of Teramo, Località Piano D’Accio snc., 64100 Teramo, Italy; 3 Istituto Zooprofilattico Sperimentale delle Venezie, Legnaro, Padova Italy; 4 Bayer Animal Health GmbH, Leverkusen, Germany

**Keywords:** *Troglostrongylus brevior*, Cat, Moxidectin, Treatment

## Abstract

**Background:**

*Troglostrongylus brevior* is a lungworm of wild felids that recently has been recognized as agent of severe respiratory disease in domestic cats in Mediterranean and Balkan countries. Nevertheless, the information on treatment options for feline troglostrongylosis is still poor. The aim of this pilot field trial was to evaluate the efficacy of the spot-on formulation containing 1% w/v moxidectin and 10% w/v imidacloprid (Advocate^®^ spot-on solution for cats, Bayer Animal Health GmbH) in the treatment of *T. brevior* infection in naturally infected cats in Greece.

**Methods:**

The trial was a negative control, multicentre, clinical efficacy study conducted according to the standards of Good Scientific Practice (GSP). Sixteen cats in two study sites, naturally infected with *T. brevior*, were allocated to an untreated control group (G1, *n* = 8) or a treatment group (G2, *n* = 8), according to a randomization list. Animals assigned to G2 were treated with Advocate^®^ for cats on days 0 and 28 at the recommended dose rate and animals assigned to G1 received a rescue treatment with the same product on days 56 and 84. Efficacy was assessed on days 28 and 56 in G2 and on days 84 and 112 in G1 by faecal larval counts. The primary efficacy criterion was the absence of *T. brevior* first-stage larvae (L1) following treatment. Other efficacy parameters were the quantitative comparison of L1 presence before (baseline) and after one or two treatments in both groups.

**Results:**

All G2 cats were negative for *T. brevior* L1 at the first post-treatment evaluation (100% efficacy) while G1 cats were persistently shedding L1. The difference of the mean number of L1 per gram between G2 and G1 was statistically significant (*P* < 0.001). All G1 cats were negative (100% efficacy) for *T. brevior* L1 at the first post-rescue-treatment evaluation. Therefore, treatment efficacy at study completion was 100% in both groups in terms of stopping the L1 shedding in the faeces of the animals. No adverse effects were observed during the study.

**Conclusions:**

These results indicate that Advocate^®^ spot-on solution for cats represents an option for treating cats naturally infected with *T. brevior*.

## Background

*Troglostrongylus brevior*, a parasitic nematode that affects bronchi and bronchioles of wild felids (e.g. *Felis silvestris silvestris*), has recently attracted scientific interest, due to its ability to induce severe bronchopneumonia in domestic cats and its apparent spreading in various countries [1]. The parasite has an indirect life-cycle involving a gastropod (terrestrial slugs or snails) as intermediate host, in which the first-stage larvae, shed by the infected cat, develop into the infective third larval stage (L3). Paratenic hosts (birds, rodents, reptiles) probably play a key role in the biological cycle, facilitating the transmission of the parasite to the definitive host [2]. A vertical route of transmission is also known although it is unclear if it occurs *via* the utero or/and the milk [3]. *Troglostrongylus brevior* infection in cats has been increasingly reported since 2010 in different countries of Europe, i.e. Spain, Italy, Greece, Cyprus and Bulgaria, with a prevalence ranging from 1.2% to 14% [4]. In the vast majority of cases, this lungworm has been described in areas where the natural host of *T. brevior*, the European wildcat, is present, although infected domestic cats have been found also in regions where the presence of wildcats is not documented [4, 5].

Cat troglostrongylosis has been proven severe and life-threatening in kittens and young animals, as it can cause irreversible lung damage and is a potential threat for the litter in the case of pregnancy or lactation [1–4, 6, 7].

To date, the only product licensed for the treatment of *T.* *brevior* infection in cats is a spot-on formulation containing eprinomectin in combination with fipronil, (S)-methoprene and praziquantel (Broadline^™^ spot-on solution for cats, Boehringer Ingelheim, Ingelheim am Rhein, Germany). Other anthelmintics containing emodepside or moxidectin have recently shown their potential effectiveness in treating *T. brevior* infection in pilot studies [8], in clinical settings [9] or in experimental studies on intermediate hosts [10]. Advocate^®^ spot-on solution for cats (Bayer Animal Health GmbH, Leverkusen, Germany) is a combination of 1% w/v moxidectin and 10% w/v imidacloprid, licensed globally for treatment and prevention of a variety of ecto- and endoparasites. In Europe, this product is labelled for feline cardio-pulmonary and intestinal nematodes for prevention (*Dirofilaria immitis*) and*/*or treatment (*Capillaria aerophila*, *Toxocara cati* and *Ancylostoma tubaeforme*) [11]. More recently it has also been labelled for prevention and treatment of *Aelurostrongylus abstrusus*. Given preliminary data that have indicated the potential utility of moxidectin in treating *T. brevior* infection in cats [9, 10], this study investigated the efficacy of Advocate^®^ against *T. brevior* in naturally infected cats in enzootic areas of Greece.

## Methods

### Study design

The study (BAH Study No.: 205121) was conducted according to the standards of good scientific practice (GSP) and the National Animal Welfare requirements, with the approval of the National Organisation of Medicines of Greece (No. 25953). The study was a negative control, multicentre, partially blinded (examining investigator blinded) trial, following a randomized block design.

### Pre-inclusion screening

Between study days (SD)-18 and SD-10, faecal samples of cats under null or irregular antiparasitic treatments for endoparasites, referred to private veterinary practices located in seven study areas, i.e. two already known as enzootic (Mykonos and Attica) [7] and five with no previous relevant information (Rhodes, Xanthi, Kavala, Thessaloniki and Syros), were subjected to qualitative Baermann’s examination. Parasite morphological features were identified using published keys [1, 12].

From the cats that were found positive for *T. brevior*, a faecal sample was collected between SD-6 and SD-3 and subjected to a quantitative Baermann test as previously described [13, 14] to confirm the infection and determine the baseline numbers of L1 larvae per gram of faeces (LPG). The identity of the L1 microscopically identified as *T. brevior* was confirmed with a species-specific PCR [6].

### Inclusion and exclusion criteria and allocation to study groups

On SD 0 the potentially enrollable cats were subjected to veterinary examination and body weighing. Only animals that satisfied the following criteria were included in the study: (i) cats shedding at least 15 LPG of *T. brevior* between SD-7 and SD 0; (ii) cats ≥ 9 weeks of age and weighing at least 1 kg; (iii) cats with no severe clinical signs at physical examination on SD 0; (iv) cats for which none of the restrictions of the product label was applicable; (v) cats without lesions in the application area (base of the neck) of the investigational veterinary product (IVP); (vi) cats for which the owner provided written owner consent form.

Animals for which the following criteria were confirmed were excluded from the study: (i) cats showing severe clinical signs compatible with *T. brevior* infection, for which it was estimated that the inclusion in the study might have permanently compromised health; (ii) pregnant or lactating cats, or cats for which mating was planned within the next 16 weeks; (iii) cats with known hypersensitivity to at least one of the ingredients of the IVP.

All cats meeting the inclusion and not meeting the exclusion criteria were included in the study and randomly allocated to two study groups, i.e. Group 1 (G1) left untreated (*n* = 11 cats) and Group 2 (G2) (*n* = 8 cats) treated with Advocate^®^.

### Anthelmintic treatment

Cats assigned to the G2 were treated twice at a monthly interval on SD 0 and SD 28/29. To generate additional efficacy information and for ethical reasons G1 cats received a rescue treatment twice at a monthly interval with Advocate^®^ on SD 56/57 and SD 83/84. All treatments were administered by the collaborating veterinarians at the recommended dose rate of ≥ 10 mg imidacloprid/kg body weight (BW) and ≥ 1.0 mg moxidectin/kg BW corresponding to ≥ 0.1 ml spot-on formulation per kg BW.

All cats were closely monitored by the veterinarian for 1 hour after each administration of the product to record and eventually treat any occurring adverse effect. The owners were advised to refer the animal to the veterinary clinic in the case of any unusual sign.

### Post-treatment evaluation

Cats of both groups were examined on SDs 28/29 and 56/57 assessing the post-treatment LPG value with by quantitative Baermann examination. Cats of G1 were additionally evaluated on SDs 83/84 and 111/112.

The primary efficacy criterion was the absence of *T. brevior* L1 on SD 28/29 and SD 56/57 (post-treatment) in G2 cats, evaluated at the quantitative Baermann examination. A secondary efficacy endpoint has been set based on the reduction of the LPG values from the pre-treatment assessment (baseline) to SD 28/29 (post-treatment collection 1) and 56/57 (post-treatment collection 2) in cats of G2, calculated as percent reduction according to the formula:

$$ {\text{Reduction }}\left( {\text{\% }} \right) = \frac{{\left( {{\text{Pre - treatment mean LPG }}{-}   {\text{Post - treatment mean LPG}}} \right)}}{\text{Pre - treatment mean LPG}} \times 100 $$

A further efficacy criterion was the LPG values reduction from baseline to SDs 83/84 and 111/112 in cats of G1, calculated as above.

The significance of the difference of the mean LPG between the two groups at each time point was assessed through analysis of variance (ANOVA), using the software SPSS for Windows, version 13.0.

## Results

### Cats and pre-treatment evaluation

Of the overall 336 cats examined for *T. brevior* L1 shedding, 19 (5.6%), i.e. 18 from Mykonos and 1 from Rhodes, were selected for the study based on the inclusion criteria.

Three of the cats initially allocated in G1, were withdrawn from the study as 2 of them were lost and 1 died in a car accident. The final study population (*n* = 16, 8 in G1 and 8 in G2) was composed of 9 (56.25%) female and 7 (43.75%) male cats with age ranging from 7 to 36 months and a weight ranging from 1.8 to 5 kg. All the animals completed the study according to the protocol, i.e. on SD 56/57 for G2 and on SD 111/112 for G1, except for one cat in G1 that was not presented for the final evaluation on SD 111/112 and therefore completed the study on SD 84.

At baseline, study cats had an average of 31.88 (standard deviation, sd = 36.247, 95% confidence interval, CI: 1.57–62.18) and 46.88 (sd = 50.351, CI: 4.78–88.97) LPG in G1 and G2, respectively (Table 1).

**Table 1:** Pre- and post-treatment larval faecal shedding of cats included in the study to evaluate the efficacy of Advocate® in the treatment of natural troglostrongylosis

Study day	Group	*n*	Mean LPG	SD	SE	95% CI	Min-Max	ANOVA
0	G1	8	31.88	36.247	12.815	1.57–62.18	15–120	*F* = 0.468; *P* = 0.505
	G2	8	46.88	50.351	17.802	4.78–88.97	15–165	
28	G1	8	20.63	7.763	2.745	14.13–27.12	15–30	*F* = 56.467; *P* < 0.001*
	G2	8	0	0	0	0	0	
56	G1	8	18.75	6.944	2.455	12.94–24.56	15–30	*F* = 58.333; *P* < 0.001*
	G2	8	0	0	0	0	0	

*Abbreviations*: G1, cats treated with Advocate on study days 56/57 and 83/84; G2, cats treated with Advocate on study days 0 and 28/29; n, number of animals included in G1 or G2; LPG, larvae per gram; CI, confidence interval; SD, standard deviation; SE, standard error; Min, minimum; Max, maximum

*Statistically significant results

### Efficacy evaluation

All of the cats enrolled in G2 were negative at the quantitative Baermann examination performed at SD 28/29 and SD 56/57. The cats enrolled in G1 were persistently infected with an average of 20.63 and of 18.75 LPG at SD 28/29 and SD 56/57 respectively (Table 1). Differences in mean LPG values were statistically significant (*P* < 0.001) at all post-treatment examinations (Table 1). All cats enrolled in G1 resulted negative at the quantitative Baermann examination performed on SD 83/84 and SD 111/112. The animal that was not presented for examination on SD 112 was however negative at SD 83. Therefore, treatment with Advocate^®^ showed an efficacy of 100% in eliminating *T.* *brevior* L1 shedding after a single treatment. No adverse effects were recorded after each administration of the product and throughout the study period.

## Discussion

The present results show that spot-on moxidectin contained in Advocate® is efficacious and safe in treating *T. brevior* infections under natural conditions.

*Troglostrongylus brevior* is the agent of a severe and emerging parasitic disease with an often fatal outcome in kittens [5, 6]. However, subclinical infections also occur [6] as in the case of cats enrolled in the present study. The diagnosis of *T. brevior* infection in cats can be challenging due to overlapping clinical features with various feline respiratory diseases, i.e. bacterial, viral or fungal infections or other parasitic diseases (i.e. aelurostrongylosis or capillariosis) [1, 6, 9]. A prompt etiological diagnosis, that would allow an immediate and effective therapeutic approach, is therefore of primary importance. Furthermore, if not treated quickly, troglostrongylosis can lead to permanent damages, such as irreversible pulmonary hypertension and related chronic complications, thus compromising the quality of life of infected cats [4, 15].

Given the emergence of *T. brevior* in cats and the subsequent increased awareness towards this parasite, the interest on the efficacy of parasiticides has increased. Apart from the licensed spot-on formulation containing eprinomectin (Broadline™, spot-on solution for cats, Boehringer Ingelheim) [16, 17] oral milbemycin oxime has also been proved potentially efficacious in treating cats infected with *T.* *brevior* either in monospecific or in mixed infection with *A.* *abstrusus*, in terms of larval shedding and complete clinical recovery [9]. However, these data were obtained from only a few cats and further investigations are needed [9]. Another trial [8] proved that the spot-on formulation containing emodepside in combination with praziquantel is highly effective against *T.* *brevior* after one (87.5%) or two (100%) administrations two weeks apart in terms of reduction of larval shedding. Additionally, in the latter study the majority of symptomatic cats recovered clinically after the first treatment, except for one of the cats for which a second administration was necessary for a complete clinical recovery [8].

Until now, spot on moxidectin has been administered in a few cats infected with *T.* *brevior*. For instance, a single administration of Advocate^®^ was 100% effective in reducing the larval shedding in two kittens with monospecific infection by *T. brevior*, with one of these kittens clinically recovering after the single spot-on application, while the other showed clinical and radiographic signs up to four weeks post-treatment before reaching a complete recovery, probably due to an initial more severe clinical condition [9]. In the same study, two repeated administrations, two weeks apart, were necessary for the elimination of larvae shed by three cats infected with both *A. abstrusus* and *T. brevior* [9]. In a study focusing on larval infectivity in mollusc intermediate hosts, spot-on moxidectin also proved highly efficacious in stopping *T. brevior* larval shedding in cats after a single administration [10]. However, in a fatal case of troglostrongylosis in a kitten, the administration of moxidectin spot-on did not succeed [18], most likely due to the general condition of the animal at admission and the severe lung lesions caused by the parasite. Thus, a prompt diagnosis and a timely and effective treatment are crucial, especially because preventive options against *T. brevior* infection are currently unavailable. Nonetheless, a certain efficacy of Advocate^®^ in preventing *T. brevior* has been recently discussed in a study that has shown that cats living in areas enzootic for *T. brevior* and subjected to known risk factors (i.e. preying, outdoor habitat) were negative for this lungworm when under a chemopreventive scheme with Advocate^®^ applied for *D. immitis* [19]. Moreover, with regard to kittens, given that Advocate® cannot be administered to cats under nine weeks of age, the application of preventive treatments of pregnant cats could potentially protect the kittens from the vertical transmission. Nevertheless, it should be taken in account that Advocate^®^ could be administered to pregnant cats only according to a benefit-risk assessment and under the supervision of a responsible veterinarian [11].

## Conclusions

The present data demonstrate the 100% efficacy of Advocate^®^ in terms of stopping larval shedding in cats naturally infected with *T. brevior*. The cessation of L1 excretion was achieved already with a single administration 28/29 days post-treatment in all study animals. Furthermore, no adverse effects were recorded in any of the cats included in the trial. Advocate^®^ acts on a variety of endo- and ectoparasites, with a broad spectrum that is advantageous especially for cats with an outdoor lifestyle. Its pharmaceutical form allows relatively easy and effective administration even to cats that are not easy to capture or handle, or to feral animals. In conclusion, Advocate^®^ appears promising for the treatment of natural infection in cats and further studies are warranted to evaluate this product also in cats with evident clinical manifestations, in order to investigate the effectiveness of one or more administrations to achieve a clinical recovery. Finally, given that Advocate^®^ can be administered monthly for the chemoprevention of *D. immitis* in cats, its ability in the prevention of troglostrongylosis is worth of further investigations.

## Data Availability

All data generated or analyzed during this study are included in this published article.

## Abbreviations

GSP: good scientific practice
G1: Group 1 (control group)
G2: Group 2 (treatment group)
L1: first larval stage
L3: third larval stage
SD: study day
IVP: investigational veterinary product
LPG: larvae per gram of faeces
ANOVA: analysis of variance
